# Development and Validation of a Method of Body Volume and Fat Mass Estimation Using Three-Dimensional Image Processing with a Mexican Sample

**DOI:** 10.3390/nu16030384

**Published:** 2024-01-29

**Authors:** Fabián Ituriel García Flores, Miguel Klünder Klünder, Miriam Teresa López Teros, Cristopher Antonio Muñoz Ibañez, Miguel Angel Padilla Castañeda

**Affiliations:** 1 School of Medicine, National Autonomous University of Mexico (UNAM), Mexico City 04510, Mexico; fgarcia8203@gmail.com; 2 Research Subdirectorate, Children’s Hospital of Mexico Federico Gómez, Dr. Marquez St. 162, Colonia Doctores, Mexico City 06720, Mexico; 3 Health Department, Santa Fe Campus, Iberoamerican University, Prol. Paseo de la Reforma, Zedec Sta Fé, Álvaro Obregón, Mexico City 01219, Mexico; miriam.lopez@ibero.mx; 4 Institute of Advanced Materials for Sustainable Manufacturing, Tecnológico de Monterrey, Canal de Miramontes, Tlalpan, Mexico City 14380, Mexico; cristopher_antonio@tec.mx; 5 Applied Science and Technology Institute (ICAT), National Autonomous University of Mexico (UNAM), Mexico City 04510, Mexico

**Keywords:** 3D cameras, Bod Pod, body composition, body volume, DXA, fat mass, Kinect

## Abstract

Body composition assessment using instruments such as dual X-ray densitometry (DXA) can be complex and their use is often limited to research. This cross-sectional study aimed to develop and validate a densitometric method for fat mass (FM) estimation using 3D cameras. Using two such cameras, stereographic images, and a mesh reconstruction algorithm, 3D models were obtained. The FM estimations were compared using DXA as a reference. In total, 28 adults, with a mean BMI of 24.5 (±3.7) kg/m^2^ and mean FM (by DXA) of 19.6 (±5.8) kg, were enrolled. The intraclass correlation coefficient (ICC) for body volume (BV) was 0.98–0.99 (95% CI, 0.97–0.99) for intra-observer and 0.98 (95% CI, 0.96–0.99) for inter-observer reliability. The coefficient of variation for kinetic BV was 0.20 and the mean difference (bias) for BV (liter) between Bod Pod and Kinect was 0.16 (95% CI, −1.2 to 1.6), while the limits of agreement (LoA) were 7.1 to −7.5 L. The mean bias for FM (kg) between DXA and Kinect was −0.29 (95% CI, −2.7 to 2.1), and the LoA was 12.1 to −12.7 kg. The adjusted R^2^ obtained using an FM regression model was 0.86. The measurements of this 3D camera-based system aligned with the reference measurements, showing the system’s feasibility as a simpler, more economical screening tool than current systems.

## 1. Introduction

In Mexico, 72% of those over 20 years old are overweight or obese, making this health issue the most prevalent in the country. Obesity is associated with diabetes, metabolic syndrome, and cardiovascular diseases and causes significant healthcare expenses [1]. Evaluations of an individual’s health consider their nutritional status [2,3], including body composition (BC), such as fat mass (FM), muscle mass, and bone mass. FM is one of the most widely studied health risk factors and is associated with cardiovascular and chronic degenerative diseases.

The percentage of body adiposity varies throughout the human lifespan. At six months, a neonate’s FM can comprise up to 30% of their BC, but this has usually decreased to 10–20% by the age of five. During adolescence, adiposity increases considerably, and from young adulthood (>20 years) onward, there is a further gradual increase, with maximum adiposity usually occurring at around the age of 50 [4]. In adults, adiposity of ≥26% in men and ≥35% in women is considered excessive [5].

In the past, methods such as cadaveric studies, hydrodensitometry (underwater weighing), deuterium dilution, and bioelectrical impedance analysis (BIA) have been used to measure BC [6]. Some densitometric methods use body volume (BV) estimation to compute BC. In 1995, air displacement plethysmography (ADP) was introduced, which uses a device known as a “Bod Pod” that estimates the individual BV from pressure oscillations using a diaphragm between two hermetic chambers inside a body capsule that the individual enters for measurement [7]. Other BV densitometric methods include magnetic resonance imaging (MRI), axial computed tomography (CT), and dual energy X-ray absorptiometry (DXA).

DXA is performed using a body scanner device that is passed over an examination table (on which the individual lies) by a movable arm. It contains a fan-shaped sensor or diode that emits high- and low-intensity energy derived from a polychromatic X-ray filter [8]. DXA can measure the BC of the whole body or specific regions. The BC aspects that it measures are the bone mineral content (BMC), bone mineral density (BMD) (BMC/area^2^), lean mass (organs, muscle, and water), FM, and visceral adipose tissue (abdominal adipose tissue differentiated from subcutaneous fat). 

DXA fat percentage measurements have a standard error of <3%. This is therefore considered a standard reference for the assessment of BC. Being smaller than MRI and CT devices, DXA devices reduce equipment and space costs while maintaining greater measurement precision than BIA, hydrodensitometry, or ADP [9,10,11].

However, precautions should be taken when using DXA as it carries some potential health risks (such as the attenuation of X-rays in pregnancy). The physical dimensions of DXA devices also limit their use to those within normal BC ranges as outliers may not comply with the measurement assumptions used by the device [12,13].

Several studies have been conducted on the use of digital image processing for BC measurement and anthropometry. However, these have been based on multiple 3D camera configurations or single cameras for 2D images and have evaluated body fat only, not other components of BC, such as fat-free mass. Other validated studies have calculated anthropometric dimensions but not BC components. The ability to calculate new anthropometric parameters using digital image processing and low-cost cameras would allow the identification of new indicators and risk factors, such as the surface area index or the trunk volume index over total BV.

Recent advances in 3D computer scanning technology allow anthropometric measurement and the estimation of BC components. Such technology includes the body scanner cabinet with several WBX 3D cameras by Cyberware [14], the Ein-Scan Pro white light sensor by CAD AVSHMEIP [15], and the Kinect sensor, which uses infrared (IR) 3D vision [16]. These devices facilitate the development of an economical, reliable, and valid method of BC measurement [17,18,19,20].

A comparative study by Tinsley et al. [21] evaluated four commercial scanner systems to assess their accuracy in estimating BV and regional anatomies. High precision was observed in different anthropometric estimations, with variable under- and overestimations depending on the anatomical region. However, despite this variability, 3D scanning technology is likely to improve rapidly and so represents a valuable practical tool for health anthropometrics, such as BC measurements, especially as 3D technologies become more accessible. Soleau et al. [18] have developed a 3D Kinect-based imaging system for anthropometric body measurements. The comparison of several anthropometric measurements estimated with the system with a reference laser system showed relative errors. Nontheless, the attempt demonstrated the feasibility of using low-cost technologies for anthropometric evaluation. Another limitation of the system by Soleau et al. was its use of an array of 16 synchronized cameras, increasing the system’s complexity and cost. This motivated research into simpler alternatives. Kennedy et al. evaluated an inexpensive commercial body scanner that used three Intel RealSense low-cost infrared cameras. The study found varying reliability for six anthropometric measurements. There were acceptable correlations with the reference DXA evaluation and the system offered a low-cost alternative to current optical systems [22].

Another study presented results from the same system but with software enhancements, including non-rigid avatar reconstruction and parameterized body model fitting. They demonstrated high reliability for body fat estimation, reinforcing the capacity of current low-cost systems for BC evaluation [23].

The latest research has used smartphone 2D images and digital image processing to assess BC in conjunction with sophisticated processing methods such as machine learning and deep learning [24,25,26]. Graybeal et al. presented an analysis method of BC measurement (FM, percentage of FM, and fat-free mass) using digital images obtained from two smartphone images. They compared the system’s precision and agreement with those of a four-compartment model that used Lohman’s equation, the international gold standard, and found a significant intraclass correlation coefficient (ICC) [26]. Digital image processing for BC evaluation has undergone segmentation refinement using new artificial neuronal network techniques. This has shown high accuracy for FM, pelvic muscles, and bone mass in pelvic axial CT images [27].

Stark et al. used an older technique called photogrammetry to reconstruct 3D body models using open-source libraries from smartphone images. Despite being an interesting approach due to the system’s facility for practical use, the study did not evaluate the precision and validity [28].

In this paper, we propose a digital image processing method for BC analysis based on a simple physical configuration using two portable, easy-to-install, economical cameras. We compare the FM estimations of our system with those of a DXA system.

## 2. Materials and Methods

### 2.1. Computer Estimation of Body Volume

**Figure 1:**
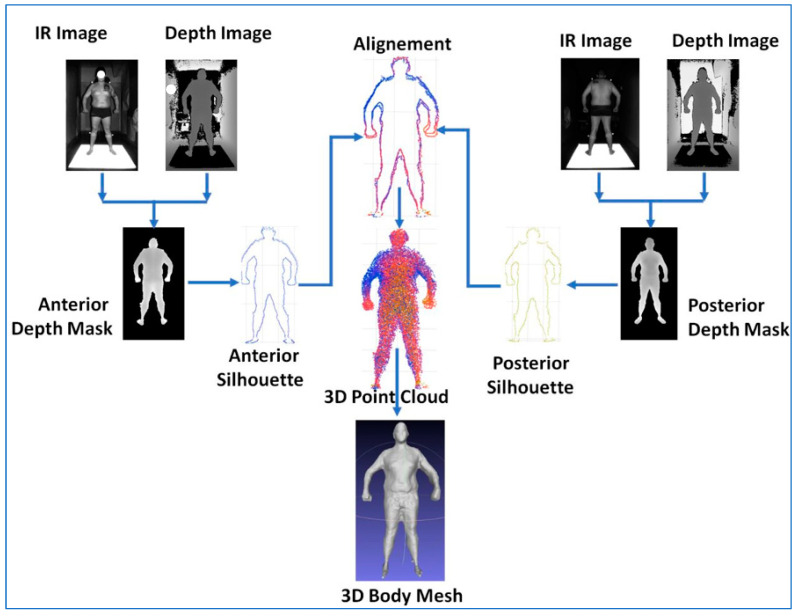
General schema of a 3D computer modeling method using two Kinect V.2 cameras to obtain anterior and posterior views for body composition analysis. IR, infrared.

The method that we propose estimates BV using a 3D computer reconstruction of the body to be evaluated. A pair of commercial stereo cameras (Kinect v.2, Microsoft, Redmond, WA, USA) [16] were arranged so that each was about 1.9 m from the body (front and back cameras); two digital images were obtained per camera, as illustrated in Figure 1. These were an IR image of the body and a depth image. In total, four images were acquired per participant.

Image segmentation based on region growth and thresholding was used to detect the body shape from the IR image, which served as a mask to isolate the depth information specific to the participant’s body from the depth image to produce a depth mask image from the 3D data of the corresponding body view. Shape and depth images were fused into anterior and posterior depth masks. The shape images helped with the selection of the corresponding depth information for the body contained in the IR images. In this way, an anterior depth mask image was used to acquire depth information from the anterior IR view of the participant. Similarly, a pair of posterior depth IR images were used to obtain the body’s posterior depth information.

A rigid platform painted with highly reflective paint was used to separate each participant’s legs and feet from the floor in the IR images to facilitate the separation of the body shape from the background.

The 3D points of the anterior and posterior silhouettes were extracted by applying a series of morphological dilatation and erosion operations to both depth mask images. Next, the silhouettes were spatially aligned using the iterative closest points algorithm, resulting in a single 3D point cloud of the participant’s whole body. With the aid of a Poisson 3D reconstruction algorithm, a 3D surface mesh of the body (3D body mesh) was created from the 3D point cloud. The mesh was filtered and smoothed to remove artifacts and geometrical imperfections. The result was the final 3D body model from which BV was calculated. 

Image acquisition was performed using the C++ programming language. This was implemented in Matlab^®^ R19 for image segmentation and alignment, while the mesh reconstruction, filtering, and volume computation were performed using MeshLab^®^ 2016.12 software scripts.

### 2.2. Experimental Study

#### 2.2.1. Objective 

This study aimed to evaluate the precision and validity of a straightforward densitometric method of BC analysis, using two commercial 3D cameras and digital image processing to estimate BV and FM. The results of the system were compared with those obtained from established methods, using ADP as the reference for BV estimation and DXA as the FM reference.

#### 2.2.2. Recruitment

The sample was obtained by direct invitation to the general public of Mexico City. The inclusion criteria were healthy men and women aged 20–40 with BMIs from underweight to obese. The exclusion criteria were pregnancy, edema, a height < 1.94 m, and a weight < 136 kg. After the study was explained to them, participants signed a written informed consent form. The study was approved by the Ethics Committee of the Children’s Hospital of Mexico Federico Gómez (protocol HIM-2017-141) and was conducted in accordance with the tenets of the 2013 revision of the Declaration of Helsinki. None of the participants were excluded from the final analysis. The researchers covered the expenses of the participants.

#### 2.2.3. Procedure

Participants were asked to fast for 6 h before the study and to maintain normal hydration levels. For the BC evaluations, they were asked to wear a swim cap, to occlude hair from the images, and a swimsuit or underwear, ensuring that it was not so tight as to change the silhouette captured by the system. Computer estimation of BV and FM using our image processing method was performed by two independent observers, who made three estimations each. Participants were asked to exhale and hold their breath for 7 s such that their lungs were at their functional residual capacity volume during measurements. In total, six measurements were made per participant.

Measurements using the system proposed in this study were performed first, followed by the reference BC measures. These were DXA (Hologic Discovery-WI, Hologic Inc., Marlborough, MA, USA) and ADP (Bod Pod, Life Measurement Inc., Concord, CA, USA).

Each participant’s air lung volume during Bod Pod (model A-661-230-023 with Bod Pod Suite software) BV measurement was adjusted as follows [7]: BV was measured at least twice, with a third measurement if the first two differed by more than 150 mL or 0.3%. The average of the two closest values was used for subsequent calculations. The thoracic gas volume was measured by instructing participants to breathe through a tube. Body density was then calculated by dividing the body mass by the corrected BV, and BF% was derived using the two-compartment Siri equation. All calculations were performed using the Bod Pod Suite software [29]. For each participant, all measurements were carried out on the same day in a single session at the Nutrition Clinic and Evaluation Center for the Elderly, Health Department, Universidad Iberoamericana, Santa Fe Campus, Mexico.

#### 2.2.4. Statistical Analysis

The Epidat 4.1 statistical software was used to calculate ICC values for intra- and interrater concordance and Bland–Altman plots were created to determine the degree of concordance between the FM measurements of the reference and experimental methods. A regression model was created using Minitab 18.

## 3. Results

The average age of the 28 participants was 28.3 (±6) years, the average BMI was 24.5 (±3.7) (23.9 for women and 26.3 for men), 60% were single, and 43% were classed as sedentary (<150 min of physical activity a week). The BMIs of 49% of the participants fell within the overweight or obese range. None of the participants were bodybuilders (Table 1).

### 3.1. Reliability and Precision of Fat Mass Prediction Using the Proposed Method

To measure the intra-observer reliability of the BV measurements obtained using our Kinect camera method, the following indices were obtained for each observer: observer 1, ICC = 0.99 (95% CI, 0.98–0.99); observer 2, ICC = 0.98 (95% CI, 0.97–0.99). For interrater reliability, the ICC was 0.98 (95% CI, 0.96–0.99). The BV error was measured as the mean difference between the Bod Pod (67.03 L) and Kinect (72.31 L) measurements, giving a raw error of 7.8% (Table 2).

The goodness-of-fit corrected the BV error through linear regression, with the Bod Pod BV measures as a reference. This gave a final error of 0.1% or 0.07 L for the fitted Kinect BV (Table 1), with a coefficient of variation of 0.20.

### 3.2. Validity of Fat Mass Prediction Using the Proposed Method

Once BV had been estimated and adjusted, a linear regression model was created for the estimation of FM. To select the best regression model, we implemented best subsets regression using the maximum R^2^ criterion and participant variables, including age, sex, body mass, height, BMI, BV, and the level of physical activity.

Multivariate analysis of the best subsets regression was performed to obtain a predictive model incorporating the participant variables. Based on the dumb rule, 5–10 samples were needed [30]. With α < 0.05, a moderate effect size of 0.5, and power of 0.60, 40 sample units were required [31]. We calculated the sample size using the GPower v. 3.1 software for a random linear multiple regression model with a priori α < 0.05, effect size R^2^ = 0.75, and power = 0.95 for four variables given 15 sample units. The analysis resulted in the following equation for FM estimation:

FM = 42.5–5.73 (sex) − 0.02254 (physical activity minutes/week) − 26.3 (height) + 0.4879 (fitted Kinect BV).(1)

where the sex variable takes the value of 1 for women and 2 for men.

**Figure 2:**
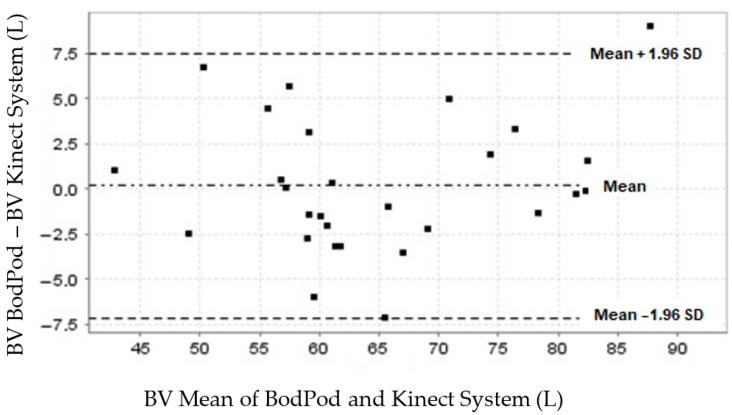
BV A plot of the concordance between body volume measurements obtained by the Bod Pod and Kinect-based systems.

The concordance between the Bod Pod and our Kinect system measurements for BV showed a mean difference (bias) close to zero (0.16; 95% CI, −1.2 to 1.6) [32,33,34] (Table 3). The normal data distribution graphically depicted by the limits of agreement (LoA) was 7.1 to −7.5 L, set at 1.96 SD (±3.7) L (Table 3). Of the data obtained from our participants, 96.4% (27/28) were within these limits (Figure 2).

**Figure 3:**
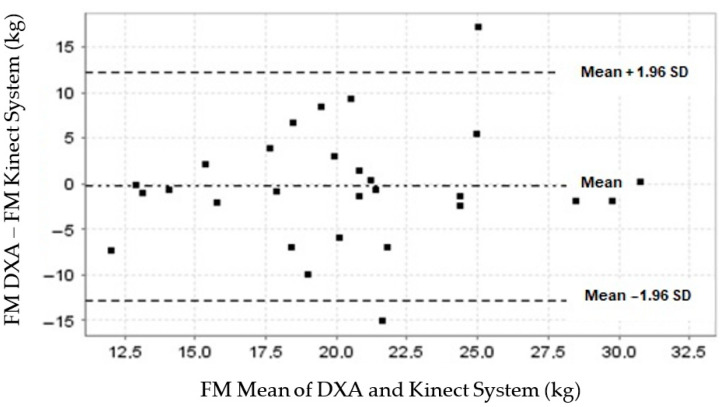
A plot of the fat mass measurement concordance between the DXA and Kinect-based systems.

The concordance between the DXA and Kinect system measurements of FM showed a mean difference (bias) close to zero (−0.29; 95% CI, −2.7 to 2.1) (Table 4). The normal data distribution graphically depicted by the LoA was 12.1 to −12.7 kg, set at 1.96 SD (±6.3) kg (Table 4). Of the data obtained from our participants, 92.8% (26/28) were within these limits (Figure 3).

The FM obtained using Equation (1) showed a coefficient estimation of adjusted R^2^ of 0.86 and Mallows’ Cp of 4.9, with a post hoc power of 0.66.

The pure error for the FM from DXA and the predicted FM from Kinect was 1.4 kg, with a variation coefficient for FM from Kinect of 0.28.

## 4. Discussion

The BV measurements obtained using the proposed method showed a consistent tendency towards slight underestimation when compared to the Bod Pod reference measurements. This was likely due to the limitations of the cameras and the lateral information from the images of participants’ bodies, which added volume to the 3D model. However, given the good reliability of the intra- and inter-observer indices, it was possible to determine the goodness-of-fit, with a BV error of 0.07 L for BV and FM estimation, with an acceptable R^2^ of 0.86 from a simple two 3D camera system.

Another advantage of this image processing system is the lack of anatomical limits in clinical assessments that hinder DXA table scans, preventing its use with those weighing less than 20 kg or more than 150 kg and those over 198 cm in height.

Xu et al. [35] achieved very good agreement between the estimated BV from 3D imaging and Bod Pod estimations, with an R^2^ of 0.9996 and an error of 0.690 l. Although precise measurements were achieved, their 3D body reconstruction was made with a complex system of up to 12 commercial cameras, making this a less practical option for use in clinical practice. Giachetti et al. [17] reported a Pearson correlation of 0.96 with acceptable agreement on Bland–Altman plots using expensive 3D commercial body scanners. Again, this is not practical for clinical applications.

Kennedy et al. [22] evaluated a 3D commercial system that measured body circumferences and FM%. The system used three vertically aligned Intel RealSense depth systems built into the aluminum frame of a standing mirror. The results were compared to those obtained by manual measurements and DXA. The reliability test–retest estimations were acceptable on both the Bland–Altman plots and regression analysis. The circumference measurements showed considerable variation (0.4–2.7%) and there were significant mean differences between measurements from the system evaluated and the reference measurements for five circumference comparisons. The correlation between methods was R^2^ = 0.73. However, the price for the hardware used by Kennedy et al. is relatively low (1400 USD). 

Some studies have used 3D cameras to calculate anthropometric measurements such as circumferences, lengths, and BV, which then become variables that feed predictive models. These approaches tend to favor the estimation of BC components such as muscle mass, bone mass, and visceral adipose tissue [36,37,38]. For this purpose, other predictive models, such as artificial neuronal networks, can be applied to improve the precision of regression models and make better approximations. More extensive image databases may pave the way for the development, training, and validation of more advanced algorithms, such as those utilizing artificial intelligence [26,39].

There was some variability in the results from our 3D cameras due to undesired artifacts and geometrical imperfections close to the arms, trunk, thighs, and pelvis junctures in the 3D images. This led to BV overestimations and was a limitation of this study. Recently, new and improved 3D cameras, such as the Intel RealSense, have become available. These range in price from 250 to 550 USD and have greater RGB resolutions (1920 × 1080), among other interesting features. These cameras could reduce image imperfections, increasing the precision and, in turn, the possible clinical applications.

Another limitation is that the layout of this system depends on the operator setup for the correct distance and alignment of the 3D camera. To implement this system in practice, a more standardized setup must be developed to minimize measurement errors due to variability in the location of the cameras due to operator misestimation or space constraints. However, the availability of new commercial cameras with more compact designs and higher resolutions than the Kinect cameras will enhance the system setup by allowing similar results to be attained in more constrained physical scenarios.

Adler et al. evaluated the % body fat and BV in 32 participants using a 3D photonic body surface scanner (VirtusSmart), with Bod Pod as the reference measure. They reported agreement results using Bland–Altman plots. For BV, the mean difference was 1.1 L, SD (±0.9) l; for % body fat, the mean difference was 7.0% SD (±5.6)%, LoA 18.1, with a −4.3% BF [20]. The study combined three different BF% results in one plot. Even when the body fat percentage was adjusted by the coefficient of FM over body weight, there was still considerable variation.

Although the data distribution between methods in the present study produced an acceptable LoA, the width of the FM LoA (up to 12.7 kg of the FM) was due to considerable SD differences (±6.3) kg. This may have been because our sample included participants in the underweight, eutrophic, overweight, and obese BMI strata, leading to greater variability in the LoA calculations. A possible strategy to minimize this issue would be the creation of separate graphs for each BMI stratum, with sufficient degrees of freedom for each stratum and a study sample of approximately 100 participants. It would also be necessary to have an independent external sample for the external validation of the results presented in this work.

Another enhancement to the system that could improve the estimation accuracy might be the incorporation of new image segmentation methods, such as machine-learning-trained methods. This would, firstly, reduce artifacts in the images, affecting the 3D reconstructions and biasing the volume estimation. Second, it would enhance the practicality of the system by facilitating the isolation of the body shape from the image background, especially at the level of the feet, thus avoiding the need for a high-reflectance platform. After this initial validation study, efforts could be directed to the development of a user-friendly interface, such as a graphical desktop interface application that quickly processes the entire measurement procedure and feeds the data back to the clinical operator.

In any case, the presented system, consisting of only two 3D cameras, is simpler than existing approaches and has demonstrated its feasibility as a tool for the estimation of FM and BV through the achievement of similar statistical results to those of other studies discussed above. Thus, while the system is not sufficiently accurate to be used as a reference method, like the Bod Pod or DXA, it is a valid screening tool for interventions such as behavioral change programs (diet, exercise) and is able to evaluate baseline differences in parameters customized to individuals and across different clinical environments.

## 5. Conclusions

Our results found the precision of BV and FM estimations obtained using the Kinect camera system to be acceptable. The concordance of these estimations with those obtained using the reference systems was adequate but could be improved. It is expected that as smaller cameras with higher definition become available, it will be possible to achieve improved results, with fewer space restrictions and fewer artifacts in the 3D reconstructions, reducing the variability in the final clinical parameters (BV and FM).

Further research is needed to investigate whether a larger prediction model that includes additional variables such as body density could be more precise than the gold standard four-compartment models, such as that of Lohman. Future studies with larger samples and broader populations should also seek to estimate other BC components, such as bone and muscle mass. This would further contribute to the possible eventual adoption of this screening tool in clinical settings.

## Figures and Tables

**Table 1:** Participants’ characteristics.

		Total n = 28 Mean ± SD (Range)	Female n = 14 Mean ± SD (Range)	Male n = 14 Mean ± SD (Range)
**Age**		28.3 ± 6 (20–42)	28.4 ± 6 (20–42)	28.3 ± 5 (20–37)
**Physical activity** **(>150 min/week)** **n (%)**		16 (57%)	10 (72%)	7 (50%)
**Height [m]**		1.65 ± 0.08	1.60 ± 0.07	1.72 ± 0.05
**Weight [kg]**		67.5 ± 12.2	61.2 ± 8.2	78.1 ± 12.9
**BMI (kg/m^2^)**		24.5 ± 3.7 (17.7–35.7)	23.9 ± 2.9 (17.7–29.1)	26.3 ± 4.6 (20.7–35.7)
	**Underweight** (17–18.4) n (%)	1 (3.5%)	1 (7%)	-
	**Eutrophic** (18.5–24.9) n (%)	13 (46%)	7 (50%)	6 (42.8%)
	**Overweight** (25–29.9) n (%)	11 (39%)	6 (42.8%)	5 (35.7%)
	**Obesity** (>30) n (%)	3 (10%)		3 (21%)
**BV-** **Bod Pod (L)**		66.5 ± 13.03	60.7 ± 13.2	69.7 ± 12.4
**Fat Mass-DXA** **(kg)**		19.63 ± 5.8	20.02 ± 6.1	19.3 ± 6.06
**Fat Mass-** **Kinect (kg)**		19.61 ± 5.5	20.00 ± 5.2	19.3 ± 6.1

BMI, body mass index; BV, body volume; DXA, dual X-ray densitometry; kg, kilogram; L, liter; m, meter; m^2^, meter squared; min, minute SD, standard deviation

**Table 2:** Measurement errors of the reference and experimental methods of body volume estimation.

BV Measurement Error	
	Bod Pod
Raw Kinect	5.28 L (7.8%)
Fitted Kinect	0.07 L (0.1%)
**Variation Coefficient of BV Measurements**	
Bod Pod	0.19
Fitted Kinect	0.20

BV, body volume.

**Table 3:** BV Concordance between body volume estimates obtained using the Bod Pod and the Kinect systems.

	Value	CI (95%)	
**Mean differences**	0.1670	−1.2873	1.6120
**SD differences**	3.7504		
**Mean − 1.96 SD**	7.1836	−9.6689	−4.6983
**Mean + 1.96 SD**	7.5176	5.0322	10.0029

CI, confidence interval; SD, standard deviation.

**Table 4:** Fat mass measurement concordance between the DXA and Kinect systems.

	Value	CI (95%)	
**Mean differences**	−0.2911	−2.7582	2.1760
**SD differences**	6.3625		
**Mean − 1.96 SD**	−12.7613	−16.9776	−8.5449
**Mean + 1.96 SD**	12.1791	7.9628	16.3954

CI, confidence interval; SD, standard deviation.

## Data Availability

The data presented in this study are available on request from the corresponding author. The data are not publicly available due to patent interests.

## References

[1] Instituto Nacional de Salud Pública (2021). National Health and Nutrition Survey on COVID-19. https://ensanut.insp.mx/encuestas/ensanutcontinua2021/doctos/informes/220804_Ensa21_digital_4ago.pdf.

[2] Haua Navarro K., Surveza Fernández A. (2010). El ABCD de la Evaluación del estado de Nutrición.

[3] Ledesma Solano J.Á., Palafox López M.E. (2015). Manual de Fórmulas y Tablas para la Intervención Nutriológica.

[4] Wadsworth M.E.J. (1993). Growth, maturation and body composition: The Fels longitudinal study 1929–1991. J. Epidemiol. Community Health.

[5] ACSM (2014). Guidelines for Exercise Testing and Prescription.

[6] Heymsfield S.B., Lohman T.G., Wang Z., Going S.B. (2007). Composición Corporal.

[7] Dempster P., Aitkens S. (1995). A new air displacement method for the determination of human body composition. Med. Sci. Sports Exerc..

[8] Siri W.R., Brozek J.H. (1961). Body composition from fluid spaces and density; analysis of methods. Techniques for Measuring Body Composition.

[9] Heymsfield S.B., Wang J., Aulet M., Kehayias J., Lichtman S., Kamen Y., Dilmanian F.A., Lindsay R., Pierson R.N., Yasumura S., Harrison J.E., McNeill K.G., Woodhead A.D., Dilmanian F.A. (1990). Dual Photon Absorptiometry: Validation of Mineral and Fat Measurements. In Vivo Body Composition Studies.

[10] Bacchi E., Cavedon V., Zancanaro C., Moghetti P., Milanese C. (2017). Comparison between dual-energy X-ray absorptiometry and skinfold thickness in assessing body fat in overweigh/obese adult patients with type-2 diabetes. Sci. Rep..

[11] Taylor A.E., Kuper H., Varma R.D., Wells J.C., Bell J.D., Radhakrishna K.V., Kulkarni B., Kinra S., Timpson N.J., Ebrahim S. (2012). Validation of Dual Energy X-ray Absorptiometry Measures of Abdominal Fat by Comparison with Magnetic Resonance Imaging in an Indian Population. PLoS ONE.

[12] Fuller N.J., Jebb S.A., Laskey M.A., Coward W.A., Elia M. (1992). Four-component model for the assessment of body composition in humans: Comparison with alternative methods, and evaluation of the density and hydration of fat-free mass. Clin. Sci..

[13] Heymsfield S.B., Ebbeling C.B., Zheng J., Pietrobelli A., Strauss B.J., Silva A.M., Ludwig D.S. (2015). Multi-component molecular-level body composition reference methods: Evolving concepts and future directions. Obes. Rev..

[14] Cyberware (2018). Scanner WBX Con Opcion de Software 2017. http://cyberware.com/products/scanners/wbx.html.

[15] CADAVSHMEIP (2018). Scanner Einscan-Pro 2017. http://www.einscan.com/.

[16] Microsoft (2018). Kinect One Sensor for Xbox Specifications. https://msdn.microsoft.com/enus/library/jj131033.aspx.

[17] Giachetti A., Lovato C., Piscitelli F., Milanese C., Zancanaro C. (2015). Robust Automatic Measurement of 3D Scanned Models for the Human Body Fat Estimation. IEEE J. Biomed. Health Inf..

[18] Soileau L., Bautista D., Johnson C., Gao C., Zhang K., Li X., Heymsfield S.B., Thomas D., Zheng J. (2016). Automated anthropometric phenotyping with novel Kinect-based three-dimensional imaging method: Comparison with a reference laser imaging system. Eur. J. Clin. Nutr..

[19] Zhang K., Zheng J., Gao C., Thomas D., Li X., Heymsfield S. (2014). Rapid-accurate anthropometric body shape assessment with low-cost novel 3D imaging system (391.2). FASEB J..

[20] Adler C., Steinbrecher A., Jaeschke L., Mähler A., Boschmann M., Jeran S., Pischon T. (2017). Validity and reliability of total body volume and relative body fat mass from a 3-dimensional photonic body surface scanner. PLoS ONE.

[21] Tinsley G.M., Moore M.L., Dellinger J.R., Adamson B.T., Benavides M.L. (2019). Digital anthropometry via three-dimensional optical scanning: Evaluation of four commercially available systems. Eur. J. Clin. Nutr..

[22] Kennedy S., Hwaung P., Kelly N., Liu Y.E., Sobhiyeh S., Heo M., Shepherd J.A., Heymsfield S.B. (2020). Optical imaging technology for body size and shape analysis: Evaluation of a system designed for personal use. Eur. J. Clin. Nutr..

[23] Tinsley G.M., Harty P.S., Siedler M.R., Stratton M.T., Rodriguez C. (2023). Improved precision of 3-dimensional optical imaging for anthropometric measurement using non-rigid avatar reconstruction and parameterized body model fitting. Clin. Nutr. Open Sci..

[24] Alves S.S., Ohata E.F., Sousa P.C., Barroso C.B., Nascimento N.M., Loureiro L.L., Bittencourt V.Z., Capistrano V.L.M., da Rocha A.R., Filho P.P.R. (2023). Sex-based approach to estimate human body fat percentage from 2D camera images with deep learning and machine learning. Measurement.

[25] Sullivan K., Hornikel B., Holmes C.J., Esco M.R., Fedewa M.V. (2022). Validity of a 3-compartment body composition model using body volume derived from a novel 2-dimensional image analysis program. Eur. J. Clin. Nutr..

[26] Graybeal A.J., Brandner C.F., Tinsley G.M. (2022). Validity and reliability of a mobile digital imaging analysis trained by a four-compartment model. J. Hum. Nutr. Diet..

[27] Hemke R., Buckless C.G., Tsao A., Wang B., Torriani M. (2020). Deep learning for automated segmentation of pelvic muscles, fat, and bone from C.T. studies for body composition assessment. Skelet. Radiol..

[28] Stark E., Haffner O., Kučera E. (2022). Low-Cost Method for 3D Body Measurement Based on Photogrammetry Using Smartphone. Electronics.

[29] Davis J.A., Dorado S., Keays K.A., Reigel K.A., Valencia K.S., Pham P.H. (2007). Reliability and validity of the lung volume measurement made by the BOD POD body composition system. Clin. Physiol. Funct. Imaging.

[30] Norman G.R., Streiner D.L. (1998). Biostatistics, The Bare Essentials.

[31] Hair J.F., Black W.C., Babin B.J., Anderson R.E. (2010). Multivariate Data Analysis.

[32] Bland J.M., Altman D.G. (1999). Measuring agreement in method comparison studies. Stat. Methods Med. Res..

[33] Bunce C. (2009). Correlation, Agreement, and Bland–Altman Analysis: Statistical Analysis of Method Comparison Studies. Am. J. Ophthalmol..

[34] Cardemil M.F. (2016). Comparison analysis and applications of the Bland-Altman method: Concordance or correlation?. Medwave.

[35] Xu B., Yu W., Yao M., Pepper M.R., Freeland-Graves J.H. (2009). Three-dimensional surface imaging system for assessing human obesity. Opt Eng..

[36] Farina G.L., Spataro F., De Lorenzo A., Lukaski H. (2016). Smartphone Application for Personal Assessments of Body Composition and Phenotyping. Sensors.

[37] Esco M.R., Holmes C.J., Sullivan K., Hornikel B., Fedewa M.V. (2021). Utilizing a Novel 2D Image Processing System for Relating Body Composition Metrics to Performance in Collegiate Female Rowers. Int. J. Environ. Res. Public Health.

[38] Samejima I., Maki K., Kagami S., Kouchi M., Mizoguchi H. (2012). A body dimensions estimation method of subject from a few measurement items using KINECT. Proceedings of the 2012 IEEE International Conference on Systems, Man and Cybernetics (SMC).

[39] Nahavandi D., Abobakr A., Haggag H., Hossny M., Nahavandi S., Filippidis D. (2017). A skeleton-free kinect system for body mass index assessment using deep neural networks. Proceedings of the 2017 (ISSE).

